# Integrating Microstructures and Dual Constitutive Models in Instrumented Indentation Technique for Mechanical Properties Evaluation of Metallic Materials

**DOI:** 10.3390/ma18174159

**Published:** 2025-09-04

**Authors:** Yubiao Zhang, Bin Wang, Yonggang Zhang, Shuai Wang, Shun Zhang, He Xue

**Affiliations:** 1 School of Mechanical Engineering, Xi’an University of Science and Technology, 58 Yanta Road, Xi’an 710054, China; yubiao.zhang@brunel.ac.uk (Y.Z.); 19105016005@stu.xust.edu.cn (S.Z.); 2 Department of Mechanical and Aerospace Engineering, Brunel University of London, Uxbridge UB8 3PH, UK; 3 Pinggao Electic Co., Ltd., Pinggao Group Co., Ltd., 22 Nanhuan Road, Pingdingshan 467001, China; 18039607419@163.com; 4 College of Mechanical Engineering, Xi’an Shiyou University, Xi’an 710065, China; shuai.wang@xsyu.edu.cn

**Keywords:** instrumented indentation technique, mechanical properties, material constitutive equation, microstructures, finite element simulation

## Abstract

Local variations in mechanical properties are commonly observed in engineering structures, driven by complex manufacturing histories and harsh service environments. The evaluation of mechanical properties accurately constitutes a fundamental requirement for structural integrity assessment. The Instrumented Indentation Technique (IIT) can play a critical role in the in-site testing of local properties. However, it could be often a challenge to correlate indentation characteristics with uniaxial stress–strain relationships. In this study, we investigated quantitatively the connection between the indentation responses of commonly used metals and their plastic properties using the finite element inversion method. Materials typically exhibit plastic deformation mechanisms characterized by either linear or power-law hardening behaviors. Consequently, conventional prediction methods based on a single constitutive model may no longer be universally applicable. Hence, this study developed methods for acquiring mechanical properties suitable for either the power-law and linear hardening model, or combined, respectively, based on analyses of microstructures of materials exhibiting different hardening behaviors. We proposed a novel integrated IIT incorporating microstructures and material-specific constitutive models. Moreover, the inter-dependency between microstructural evolution and hardening behaviors was systematically investigated. The proposed method was validated on representative engineering steels, including austenitic stainless steel, structural steel, and low-alloy steel. The predicted deviations in yield strength and strain hardening exponent are broadly within 10%, with the maximum error at 12%. This study is expected to provide a fundamental framework for the advancement of IIT and structural integrity assessment.

## 1. Introduction

Accurate evaluation of mechanical properties, particularly plastic behavior, is essential for assessing the structural integrity of important mechanical structures. These properties directly affect the safe operation of such structures [1]. However, critical components, such as pressure vessels, welded joints, and long-distance pipelines, often undergo complex local variations in material properties during manufacturing. Furthermore, harsh service conditions-including high temperatures, pressures, and electrochemical corrosion-can accelerate the degradation in structural materials [2,3]. As a result, the mechanical properties within a structure can become highly heterogeneous, influencing both its load-bearing capacity and failure behavior [4]. Conventional mechanical property evaluations are typically performed under uniaxial loading and require the extraction of components to prepare standardized specimens. These methods face significant limitations when applied to in-service structures, where preserving structural integrity is essential. Therefore, to improve the efficiency of structural integrity assessments and service life predictions, there is an urgent need for advanced in situ and non-destructive techniques capable of accurately evaluating local mechanical properties.

In recent years, substantial progress has been made in evaluating local mechanical properties, particularly through the use of the Instrumented Indentation Technique (IIT). Notably, the Instrumented Indentation Technique (IIT), a non-destructive method, has demonstrated strong correlation with standardized mechanical testing methods [5,6]. As a non-destructive method, IIT has demonstrated strong correlation with standardized mechanical testing methods and enables in situ evaluation of in-service structural materials. It is increasingly recognized as a key technique for characterizing mechanical behavior at the micro- and nanoscale [7], especially in heterogeneous materials such as welded structures. However, a major challenge of IIT is quantitatively correlating indentation characteristics with uniaxial stress–strain relationships. Currently, the most widely used method for evaluating elastic properties is the Oliver–Pharr approach [8]. However, for plastic properties such as yield strength and strain hardening exponent, although various methods have been proposed, the topic remains under debate within the research community. Studies by Tabor et.al [9,10,11,12,13] have shown that the stress induced by indentation is approximately 1.1 times the uniaxial mean stress at the onset of yielding, and increases to about 2.8 times under fully plastic deformation. Correlating mechanical responses precisely between indentation tests and uniaxial tensile experiments remains challenging, primarily due to the difficulty in precisely capturing the subtle surface deformations of the specimen induced by the indenter. With technological advancements, numerical simulation has been widely applied in IIT research. Numerical methodologies offer distinct advantages enabling sensitivity analysis of multiple influencing factors, thus reducing experimental uncertainties and methodological limitations. Energy-based models have been effectively implemented in simulations to establish fundamental relationships between indentation mechanics and material properties [14,15,16]. Expanding on single loading-unloading indentation tests, Haggag et al. [17] proposed a cyclic indentation method to evaluate true stress–strain behavior. This method utilizes the transition points between loading and unloading to derive the uniaxial stress–strain curve [18,19]. Some researchers have also applied this method to single loading-unloading indentation tests, aiming to directly extract the uniaxial stress–strain curve from the loading segment of the load–depth curve [20].

Although IIT has seen significant progress, current methodologies still face notable limitations. Some methods rely heavily on the unloading stage of the load–depth curve, such as the energy-based method and the continuous indentation technique. Detecting deformation during the unloading segment requires high-precision equipment, making in situ testing more complex. In contrast, data collected during the loading phase tend to be more stable and reliable. However, there is a lack of comprehensive IIT based on the loading segment. Furthermore, the continuous indentation technique yields only a partial stress–strain curve and cannot capture the full plastic deformation stage. Establishing the relationship between indentation responses and mechanical properties depends on the use of appropriate constitutive models, including the Ludwik model [21], Hollomon equation [22], and Ramberg-Osgood model [23]. Yet, the reliability of IIT-based predictions is directly influenced by how well these models represent the true stress–strain curve. Unfortunately, these equations often fail to accurately represent both small- and large-strain stages of the true stress–strain curve. Experimental observations reveal distinct hardening behaviors: austenitic stainless steels typically show linear hardening during plastic deformation, while certain alloy steels more closely follow power-law hardening. Given the complex microstructure of metallic materials, applying a single constitutive equation to all hardening behaviors is theoretically unsound. Integrating the IIT with microstructures and developing methods compatible with multiple constitutive models is essential for extending the universality of indentation theory and improving the accuracy of mechanical property predictions.

This study focuses on commonly used materials in important mechanical structures. By accounting for microstructural features and constitutive equations, it aims to enhance the applicability and accuracy of IIT across a wide range of metals, particularly addressing the significant prediction errors observed in certain alloys due to variations in hardening response. These materials exhibit different plastic deformation, characterized by either linear or power-law hardening. A single constitutive model lacks the flexibility to adequately capture such variations. Therefore, both the Hollomon (power-law) and linear hardening constitutive equations were incorporated in the material model. We investigated the relationship between the loading segment of the load–depth curve and plastic mechanical properties using a finite element inversion method. Based on analyzing the microstructures of materials with different hardening behaviors, we established the methods for predicting mechanical properties of both power-law and linear hardening materials, respectively. A systematic criterion was proposed to classify hardening behaviors based on microstructure, facilitating the selection of appropriate IIT methods. By focusing on the loading segment curve, the proposed method significantly enhances the practicality and applicability of IIT in engineering evaluations. The accuracy of this method was validated via tests on various materials, including austenitic stainless steel, structural steel, and low-alloy steel.

## 2. Methodology

**Figure 1:**
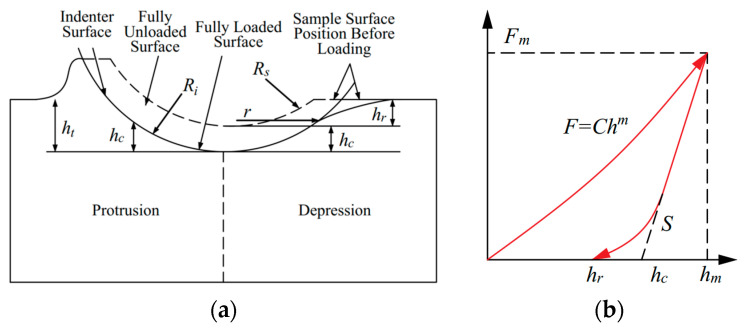
Indentation method. (**a**) Indentation response and profile geometry schematic diagram. (**b**) Typical load–depth curve.

The indentation test generally comprises two stages: the loading and unloading processes. During loading, the indenter presses into the specimen surface, initially causing elastic deformation in the contact region. As the load increases, the region experiencing the greatest elastic deformation begins to yield, entering the elastoplastic stage [24]. Figure 1a illustrates a schematic of the indentation profile created by a spherical indenter. Material parameters such as hardness and elastic modulus are typically derived using the Oliver-Pharr method. The typical load–depth curve from the indentation test is shown in Figure 1b. For a given indenter geometry, the unloading stiffness determined from the unloading segment:

(1) $$S={\frac{dF}{dh}/}_{h=h_{m}}$$

where *F* is the indentation load, *h* the indentation depth, *S* the unloading stiffness, and *h_m_* the maximum indentation depth. Further, the true contact depth and contact area can be calculated using the following formulas:

(2) $$h_{c}=h_{m}-\lambda \frac{F}{S}$$

(3) $$A=2\pi rh_{c}$$

where *h_c_* is the true contact depth, *S* the unloading stiffness in Equation (1), *A* the contact area, *r* the contact radius, and *λ* the constant related to the geometry of the indenter. For a spherical indenter, *λ* typically assigned a value of 0.75. By combining the above formulas, the expression for calculating material hardness can be derived as follows:

(4) $$H=\frac{F}{A}$$

where *H* is the hardness of the spheroidal indentation.

In the elastic contact theory, the influence of microstructural orientation, specimen dimensions, and boundary conditions is typically ignored. The specimen’s cross-sectional dimensions are assumed to be much larger than the maximum indentation depth. For isotropic materials, the specimen surface is considered as a geometric plane, leading to:

(5) $$E_{IT}=\frac{1-\nu^{2}}{\frac{1}{E_{r}}-\frac{1-\nu_{i}^{2}}{E_{i}}}$$

where *E*_i_ and *ν*_i_ represent the elastic modulus and Poisson’s ratio of the indenter material (typically diamond with values of 1140 GPa and 0.07, respectively). Furthermore, research indicates that there is a relationship between the elastic contact stiffness and the contact area as follows [25]:

(6) $$E_{r}=\frac{\sqrt{\pi }}{2\beta }\frac{S}{\sqrt{A}}$$

where *E_r_* represents the effective elastic modulus, and *β* is a geometric parameter associated with the indenter shape, which is 1.0 for a spherical indenter. With the aid of this equation, the elastic modulus and Poisson’s ratio of the material can be determined.

Metallic materials experience elastic then elastoplastic deformation under external mechanical loading. The linear relationship between stress and strain in the elastic range becomes progressively nonlinear as the material enters the plastic deformation stage. Most metals exhibit power-law hardening during plastic deformation, which is commonly described by Ludwik and Hollomon equations [26,27,28]. However, engineering materials commonly used in pipelines and welding, such as austenitic stainless steel and nickel-based alloys, often display quasi-linear hardening behavior during plastic deformation. Applying the power-law hardening models to these materials often results in significant prediction errors. Although several studies have proposed modified equations or improved constitutive models to overcome these limitations, most introduce additional parameters, making the equations excessively complex [29,30,31]. This computational complexity significantly increases the difficulty of solutions and limits practical engineering applications. Therefore, in this study, both the Hollomon power-law and a linear hardening constitutive equation were employed in finite element analysis (FEA) as material models to establish IIT. The simulation results were validated through experimental tests.

## 3. Finite Element Model

In this study, the true stress–strain curves of materials were characterized using both the Hollomon power-law and the linear hardening equation, respectively:

(7) $$\left\{\begin{array}{c} \sigma =E\varepsilon ,\left(\varepsilon \leq \varepsilon_{y}\right)\\ \sigma =E\varepsilon_{y}^{1-n}\cdot \varepsilon^{n},\left(\varepsilon >\varepsilon_{y}\right) \end{array}\right.$$

(8) $$\left\{\begin{array}{c} \sigma =E\varepsilon ,\left(\sigma \leq \sigma_{0}\right)\\ \sigma =\left(1-\delta \right)\sigma_{0}+\delta E\varepsilon ,\left(\sigma >\sigma_{0}\right) \end{array}\right.$$

where *E* is the elastic modulus, *n* the strain hardening exponent, *ε_y_* the yield strain, *δ* the reduction coefficient, and *σ*_0_ the yield stress.

Table 1 summarizes the material parameters used in the finite element inversion analysis. For both hardening models, five yield stresses were chosen, together with five different hardening parameters, respectively, to represent materials of different mechanical properties, resulting in 25 inversion simulations (5 × 5) for each hardening model. It is important to note that this range covers most materials commonly used in engineering structures. However, applying the proposed method beyond this range, particularly to high-strength or ultra-high-strength alloys, may result in reduced predictive accuracy.

**Figure 2:**
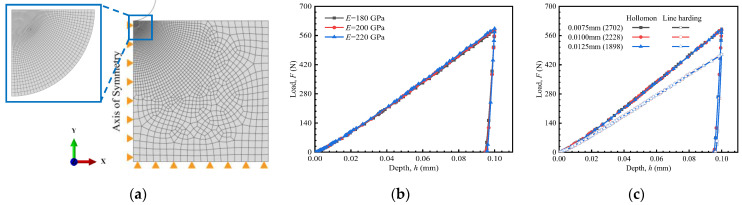
Finite element model: (**a**) FE model, (**b**) influence of *E* values, (**c**) verification of mesh independence and convergence.

The indentation model, shown in Figure 2a, consists of an indenter and a specimen. This study employs a two-dimensional axisymmetric model. This model represents a three-dimensional body with rotational symmetry about the *Y*-axis, as many previous studies have demonstrated that it enables a balance between computational accuracy and efficiency. Due to the negligible deformation of the indenter during loading, it was modeled as a rigid spherical body with a 0.5 mm radius. The specimen was modeled with dimensions of 3 mm × 3 mm. According to references [32,33], the specimen thickness-to-contact diameter ratio should exceed 15. Additionally, the distance from the indentation center to the specimen edge should be at least 4.5 times the indentation diameter. The finite element models and specimens in this study satisfy both criteria. A friction coefficient of 0.15 was applied between the indenter and specimen [34]. In simulation, initial and boundary conditions were set to match those of the indentation tests. The simulation assumed a room-temperature environment with no pre-existing residual stress or other applied loads. The bottom of the model was fixed to prevent movement in the *Y*-axis, allowing deformation only on the top surface and interior. The model’s axis of symmetry is constrained to prevent movement in the *X*-axis. The triangles in the figure simply represent these boundary constraints. The indenter is allowed to move only along the *Y*-axis, with a prescribed displacement of 0.1 mm. The reaction force and displacement at the indenter were used as the analysis outputs, which correspond to the data collected by the experimental sensors.

Numerical simulations were conducted using ABAQUS 6.14. The global model incorporates consists of eight-node biquadratic axisymmetric quadrilateral (CAX8) elements. A structured mesh with the minimum element size of 0.01 mm was applied in the central region of the indentation, while a transitional mesh was used in the adjacent area to gradually increase element size. Outside the region of interest, a free mesh with an element size of 0.2 mm was employed. The mesh independence and convergence test were performed to ensure result consistency, as shown in Figure 2c.

The elastic modulus of most steels commonly used in engineering structural components typically ranges from 190 to 220 GPa [35]. Potential influence of the elastic modulus was also investigated to illustrate possible impact on the load–depth curve using Hollomon model. As illustrated in Figure 2b, simulation results indicate that the effect of elastic modulus is negligible, in agreement with [36]. Therefore, the elastic modulus of the value of 200 GPa was used in all numerical simulations.

## 4. Experimental Procedure

Samples of six different grades of steel were used in the experimental study to develop an IIT that integrates microstructures and material constitutive models. The materials tested were stainless steel 316L and 304, low-alloy steel SA508, structural steel 42CrMo and Q235, and pipeline steel X80.

The test procedure consists of a simple tensile test and an indentation cycle involving two sequential stages: pressing the indenter into the specimen to a depth of 0.1 mm, followed by unloading to the initial position.

**Figure 3:**
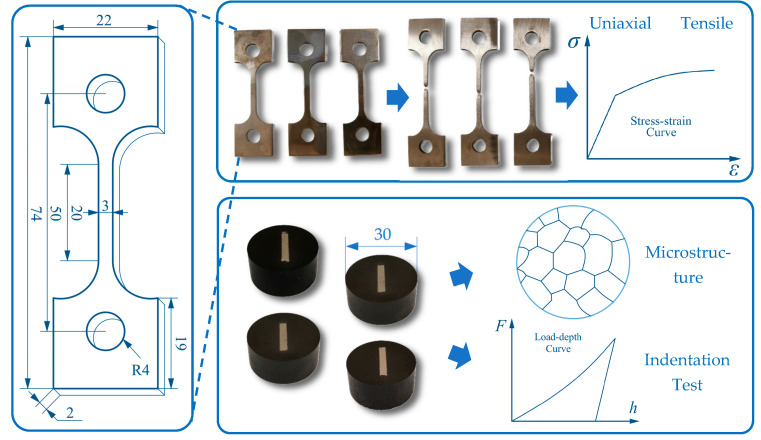
Summary of experimental processes and specimens in this study (mm).

The experimental procedure is illustrated in Figure 3. The chemical compositions of tested specimens are listed in Table 2. All materials were provided and machined into specimens for testing and prepared for microscopy observation.

### 4.1. Microstructure Inspection

Specimens underwent mosaicing, grinding, polishing, and etching procedures. Appropriately sized specimen blocks, obtained through wire cutting, were placed on the platform of the mosaicing machine with the observed plane facing downwards. After mounting, the specimens were subjected to grinding and polishing. Grinding was performed using the YMPZ-1 metallographic grinding (Chongqing Science and Technology Experimental Instrument Co., Ltd., Chongqing, China) and polishing machine to achieve a flat observation surface. Sandpapers with grit sizes of 600#, 1000#, 1500#, and 2000# were used sequentially. Polishing continued until scratch-free, mirror-like surface was obtained. The specimens were then etched with specific reagents to reveal their microstructures. Among them, SS304 and SS316L were etched with aqua regia. The remaining materials were etched with 4% nitric alcohol solution. After etching, the specimens were rinsed and dried. Finally, microstructural observations were conducted using a DM2700M optical microscope (Leica Microsystems, Wetzlar, Germany).

### 4.2. Uniaxial Tensile Test

Uniaxial tensile tests were conducted on specimens to obtain stress–strain curves and determine mechanical properties. Initially, the steel sheets were rolled into a 2 mm-thick plates by the veneer reeling machine. A wire cutting machine was then used for precision cutting, burr removal, and pinhole treatment, following EN 10088-2:2014 [37] and GB/T 24511-2009 standards [38]. The materials underwent solution treatment, annealing, acid pickling, and finishing processes before delivery. After specimen formation, heat treatment was applied to minimize internal stresses and reduce their influence on subsequent tests.

**Figure 4:**
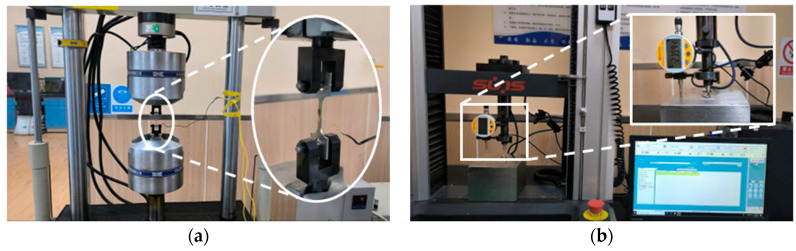
Experimental procedures and equipment: (**a**) uniaxial tensile tests on PLD-500kN testing machine; (**b**) indentation test and improved UTM2000 electronic universal testing machine.

As shown in Figure 4a, uniaxial tensile tests were conducted utilizing a PLD-500kN testing machine (Xi’an LETRY Materials Testing Technology Co., Ltd., Xi’an, China), in accordance with ISO 6892-1: 2019 [39]. Each material was tested at least three times to reduce experimental error and ensure result reliability.

### 4.3. Indentation Test

The indentation tests were conducted using the improved UTM2000 electronic universal testing machine (Shenzhen Sansi Zongheng Technology Co., Ltd., Shenzhen, China), as depicted in Figure 4b. The indentation specimens were cut from uniaxial tensile specimens to ensure consistency across all tests. A high-precision displacement sensor, with 0.5% full-scale accuracy and 0.1 μm resolution, was installed beside the indenter. A spherical indenter with a 0.5 mm radius and ±1 μm dimensional accuracy was used. The loading protocol followed quasi-static displacement control at 0.1 mm/min, with the indentation depth fixed at 0.1 mm, in accordance with ISO 14577-1 standard. Each material was tested at least three times under identical environmental conditions to ensure repeatability.

## 5. Results and Discussion

### 5.1. Influence of Plastic Parameters on Indentation Responses

To develop a method for predicting the plastic deformation stage of uniaxial stress–strain curves from the load–depth curve, the research was structured into three sequential steps. First, the influence of plastic parameters on the indentation response was analyzed in Section 5.1. Next, appropriate inversion formulas for predicting mechanical properties were established in Section 5.2 and Section 5.3. Finally, the proposed methods were calibrated and validated in Section 5.4 and Section 5.5.

**Figure 5:**
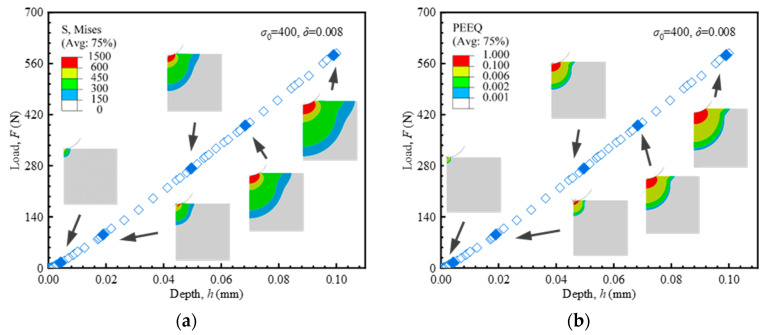
Load–depth curves from indentation tests and corresponding stress distributions from FEA (Hollomon model, *σ*_0_ = 400 MPa, δ = 0.008): (**a**) Mises stress; (**b**) PEEQ.

The Mises stress and equivalent plastic strain (PEEQ) during the indentation process were calculated and analyzed, providing a visual representation of the mechanical field. Similar mechanical field distributions were observed for both the Hollomon and the linear hardening material models. Representative data and contour plots were extracted from the finite element results of the linear hardening model. As depicted in Figure 5a, the Mises stress beneath the indenter gradually increases with increasing compression. Unlike uniaxial tension, the specimen under indentation experiences plastic deformation from the very early stage. In the initial stage, the plastic zone is surrounded by the elastic stress field. The elastoplastic transition occurs at shallow indentation depths. The evolution of the plastic zone is illustrated in Figure 5b. The transition to the fully plastic stage is marked by the plastic zone reaching the material surface. At this point, the load–depth curve is predominantly governed by plastic deformation. This confirms the feasibility of extracting plastic parameters from the loading segment.

### 5.2. Establishment of Mechanical Properties Acquisition Method Based on the Hollomon Model

At the same indentation depths, different plastic parameters of the sample materials lead to different loading forces, yielding in higher or lower load–depth curves. However, it is essential to note that the indentation load is a transient parameter and can be affected by measurement uncertainties under practical conditions. Here we introduce an empirical formula in a power-law form as Equation (9) to fit the loading segment of the load–depth curve.

(9) $$F=C^{H}h^{m^{H}}$$

where *F* is the indentation load, *h* the indentation depth, *C^H^* the loading curvature in the Hollomon model, and *m^H^* the loading exponent. The superscript *H* indicates that the Equation (9) is obtained based on the Hollomon model. The indentation response parameters obtained from the entire loading segment are more reliable than the transient parameters represented by the maximum load. They help minimize operational errors and more accurately reflect the influence of plastic properties on the load–depth curve.

**Figure 6:**
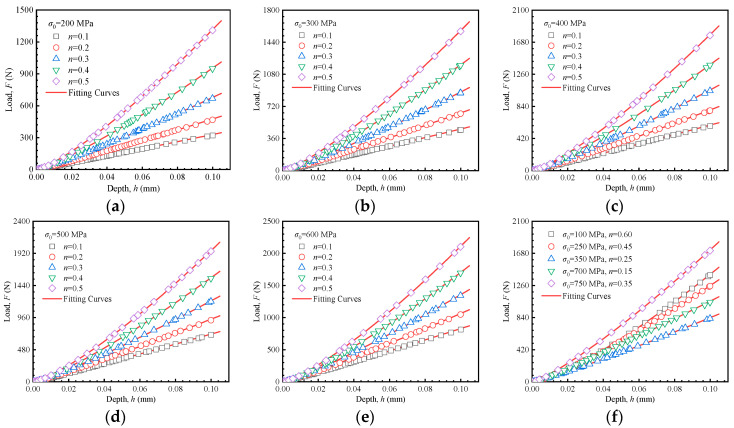
Load–depth curves of Hollomon model by FEA. (**a**) *σ*_0_ = 200 MPa, (**b**) *σ*_0_ = 300 MPa, (**c**) *σ*_0_ = 400 MPa, (**d**) *σ*_0_ = 500 MPa, (**e**) *σ*_0_ = 600 MPa, (**f**) data utilized for initial validation of predictive formula.

Equation (9) establishes a quantitative relationship among the loading curvature, the loading exponent, and mechanical properties as shown in Figure 6. The 25 sets of curves labeled (a) to (e), based on the material parameters in Table 1, constitute the dataset for developing predictive formulations of mechanical properties. The curves in (f) are independent of the data set (25 inversion simulations in Table 1) used in the inversion process. Instead, they were used solely for the preliminary verification of the subsequently established inversion formulas. Higher yield stress and strain hardening exponent lead to an increased maximum load and a steeper slope in the loading segment. This reflects a greater degree of material hardening and an increased resistance to plastic deformation. These observations form the basis of the methodology proposed in this study. 

**Figure 7:**
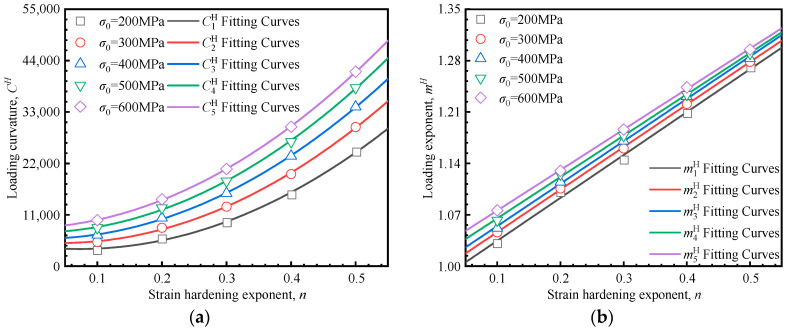
Relationship between indentation parameters and material mechanical parameters: (**a**) strain hardening exponent vs. loading curvature; (**b**) strain hardening exponent vs. loading exponent.

**Figure 8:**
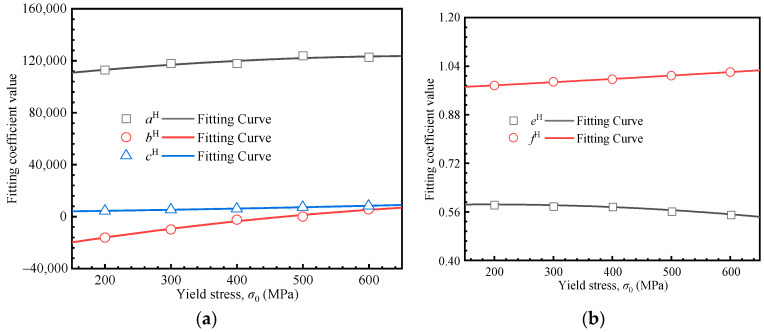
Relationship between the fitting coefficients and the yield stress: (**a**) *a^H^*, *b^H^*, and *d^H^*; (**b**) *e^H^* and *f^H^*.

To establish the relationship between the indentation responses and material mechanical properties, a more detailed investigation of the interactions amongst the yield stress *σ*_0_, the strain hardening exponent *n*, the loading curvature *C^H^*, and the loading exponent *m^H^* is necessary. The 25 load–depth curves in Figure 6a–e were fitted according to Equation (9), with the fitting results (*C^H^* and *m^H^*) presented in Figure 7. Each data point in Figure 7 corresponds to one of the 25 parameter sets used in the finite element inversion. The fitted line illustrates the relationship between *C^H^*/*m^H^* and strain hardening exponent at a constant yield strength. It can be observed that there is a quadratic relationship between the loading curvature *C^H^* and the strain hardening exponent *n*, while a linear relationship exists between the loading exponent *m^H^* and the strain hardening exponent *n*. These relationships are quantitatively described by Equation (10):

(10) $$\left\{\begin{array}{c} C^{H}=a^{H}n^{2}+b^{H}n+d^{H}\\ m^{H}=e^{H}n+f^{H} \end{array}\right.$$

where *a^H^*, *b^H^*, *d^H^*, *e^H^*, and *f^H^* represent the fitting coefficients. As shown in Figure 8, these coefficients exhibit a strong correlation with the yield stress, and their corresponding values are summarized in Table 3.

(11) $$\left\{\begin{array}{l} a^{H}=-0.043{\sigma_{0}}^{2}+59.966\sigma_{0}+102,776.143\\ b^{H}=-0.046{\sigma_{0}}^{2}+90.175\sigma_{0}-32,503.050\\ d^{H}=5.37\times 10^{-3}{\sigma_{0}}^{2}+5.490\sigma_{0}+2959.154\\ e^{H}=-1.88\times 10^{-7}{\sigma_{0}}^{2}+6.93\times 10^{-5}\sigma_{0}+0.578\\ f^{H}=1.84\times 10^{-8}{\sigma_{0}}^{2}+9.46\times 10^{-5}\sigma_{0}+0.957 \end{array}\right.$$

The simultaneous solutions of Equations (10) and (11) lead to the predictive formulation for material mechanical properties based on the Hollomon model.

(12) $$\left\{\begin{array}{c} C^{H}=\left(-0.043{\sigma_{0}}^{2}+59.966\sigma_{0}+102,776.143\right)n^{2}+\left(-0.046{\sigma_{0}}^{2}+90.175\sigma_{0}-32,503.050\right)n+5.37\times 10^{-3}{\sigma_{0}}^{2}+5.490\sigma_{0}+2959.154\\ m^{H}=\left(-1.88\times 10^{-7}{\sigma_{0}}^{2}+6.93\times 10^{-5}\sigma_{0}+0.578\right)n+1.84\times 10^{-8}{\sigma_{0}}^{2}+9.46\times 10^{-5}\sigma_{0}+0.957 \end{array}\right.$$

**Figure 9:**
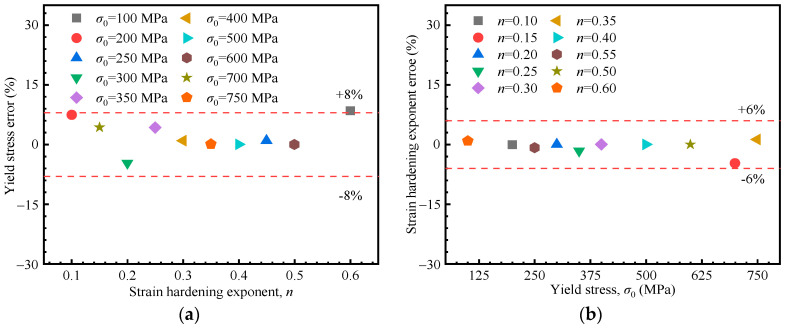
Accuracy of the method based on Hollomon model: (**a**) errors in the yield stress in terms of the strain hardening component; (**b**) errors in the strain hardening exponent in terms of the yield stress.

Using the proposed formulations, the stress–strain curve based on the Hollomon equation can be directly constructed from the loading curvature and loading exponent extracted from the load–depth curve. To evaluate the accuracy of the proposed formulation, five material parameter sets were selected from the 25 simulation cases for verification. Additionally, five additional materials not included in the inversion process were randomly selected for prediction (from Figure 6f). Verifications were conducted by comparing the results of FE simulations with those predicted by the proposed formulation. The relative errors of the yield stress and the strain hardening exponent are depicted in Figure 9, broadly within 8% and 6%, respectively, confirming the accuracy of the proposed method.

### 5.3. Establishment of Mechanical Properties Acquisition Method Based on Linear Hardening Model

**Figure 10:**
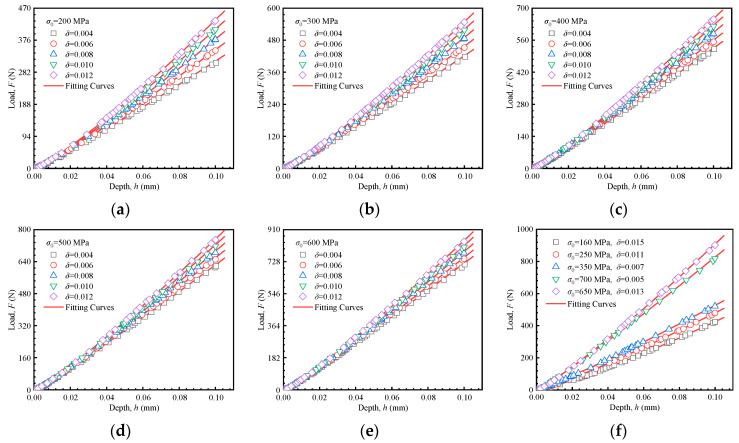
Load–depth curves of the linear hardening model. (**a**) *σ*_0_ = 200 MPa, (**b**) *σ*_0_ = 300 MPa, (**c**) *σ*_0_ = 400 MPa, (**d**) *σ*_0_ = 500 MPa, (**e**) *σ*_0_ = 600 MPa, (**f**) data utilized for initial validation of predictive formula.

**Figure 11:**
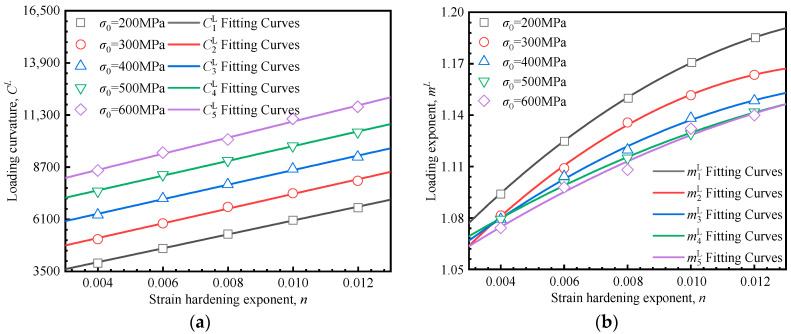
Relationship between indentation parameters and material mechanical parameters. (**a**) Loading curvature and (**b**) loading exponent vs. in the linear hardening model, respectively.

In parallel to the approach for predicting the mechanical properties using the Hollomon model, formulations based on the linear hardening model were also developed. Figure 10a–e shows the results of 25 inversion simulations along with their corresponding fitted curves. The curves dataset in Figure 10f, based on material parameters from Table 1, was used for preliminary verification of the proposed formulations. Consistent with the previous methodology, the loading segments of the load–depth curves for the linear hardening model were fitted using Equation (13).

(13) $$F=C^{L}h^{m^{L}}$$

where *C^L^* is the loading curvature, and *m^L^* the loading exponent. The superscript *L* indicates the linear hardening model. The 25 load–depth curves in Figure 10a–e were fitted according to Equation (13), and the corresponding yield stresses, strain hardening exponents, loading curvatures and exponents (*C^L^* and *m^L^*) are presented in Figure 11. Each data point in Figure 11 corresponds to one of the 25 sets of inversion parameters. The fitted line illustrates the relationship between *C^L^*/*m^L^* and strain hardening exponent under constant yield strength.

In contrast to the Hollomon model, the loading curvature exhibits a linear relationship with the strain hardening exponent, whereas the loading exponent follows a quadratic relationship with the strain hardening exponent, as shown in Equation (14).

(14) $$\left\{\begin{array}{c} C^{L}=a^{L}n+b^{L}\\ m^{L}=d^{L}{n^{2}+e}^{L}n+f^{L} \end{array}\right.$$

where *a^L^*, *b^L^*, *d^L^*, *e^L^*, and *f^L^* represent the fitting coefficients. The fitted values are listed in Table 4.

**Figure 12:**
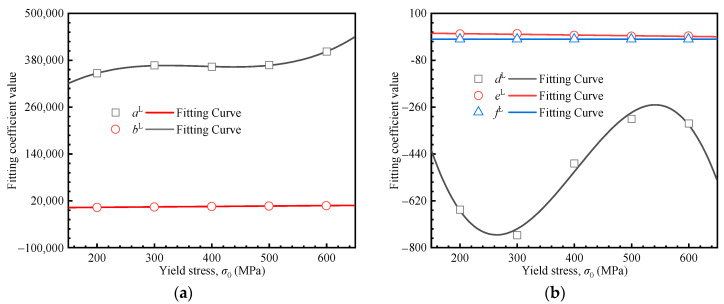
Fitting coefficients vs. yield stresses. (**a**) *a^L^*, *b^L^*, and *d^L^*, (**b**) *e^L^* and *f^L^*.

The corresponding data are shown in Figure 12. Notably, compared to the Hollomon model, the relationship between the fitting coefficients and the yield stress is more complex. Specifically, coefficients *a*^L^ and *d*^L^ require cubic fitting, whereas coefficients *b*^L^, *e*^L^, and *f*^L^ show linear correlations. The fitted equations are presented in Equation (15).

(15) $$\left\{\begin{array}{c} a^{L}=4.490\times 10^{-3}{\sigma_{0}}^{3}-5.126{\sigma_{0}}^{2}+1903.696\sigma_{0}+135152.451\\ b^{L}=11.156\sigma_{0}+366.037\\ d^{L}=-4.692\times 10^{-5}{\sigma_{0}}^{3}+0.057{\sigma_{0}}^{2}-20.141\sigma_{0}+1476.036\\ e^{L}=-0.027\sigma_{0}+27.891\\ f^{L}=4.784\times 10^{-5}\sigma_{0}+1.002 \end{array}\right.$$

Simultaneous solutions of Equations (14) and (15) yield predictive formulations for material mechanical properties based on the linear hardening model.

(16) $$\left\{\begin{array}{c} C^{L}=\left(4.490\times 10^{-3}{\sigma_{0}}^{3}-5.126{\sigma_{0}}^{2}+1903.696\sigma_{0}+135,152.451\right)n+11.156\sigma_{0}+366.037\\ m^{L}=\left(-4.692\times 10^{-5}{\sigma_{0}}^{3}+0.057{\sigma_{0}}^{2}-20.141\sigma_{0}+1476.036\right)n^{2}+\left(-0.027\sigma_{0}+27.891\right)n+4.784\times 10^{-5}\sigma_{0}+1.002 \end{array}\right.$$

**Figure 13:**
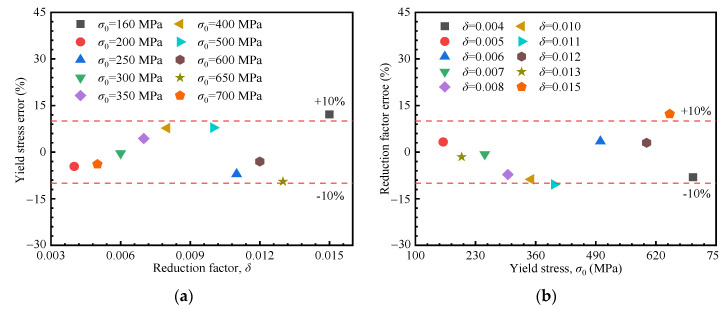
Relative errors based on the linear hardening model. (**a**) Yield stress vs. reduction factor, (**b**) Reduction factor vs. yield stress.

Using the proposed formulations, the stress–strain curve based on the linear hardening model can be derived from the loading curvature and loading exponent extracted from the load–depth curve. Preliminary validation was conducted using FE simulations. Five material sets from the inversion process, along with five additional sets excluded from inversion (shown in Figure 10f), were selected for verification. The relative errors of the yield stress and the strain hardening exponent are depicted in Figure 13, mostly within 10%, and 12% at maximum, confirming the accuracy of the proposed method.

### 5.4. Microstructure and Uniaxial Stress–Strain Curve

**Figure 14:**
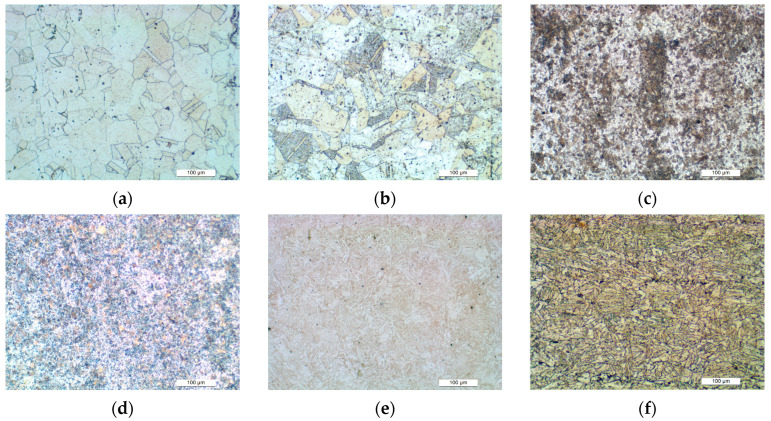
Microstructures of materials. (**a**) SS316L, (**b**) SS304, (**c**) SA508, (**d**) 42CrMo, (**e**) Q235, (**f**) X80.

Figure 14 illustrates the microstructures of six alloys: SS304, SS316L, SA508, 42CrMo, Q235 and X80. Figure 14a,b reveal that both SS304 and SS316L show a predominant austenite phase with observable recrystallized grains and ferrite precipitates. This microstructural contributes to the excellent plastic deformability of austenitic stainless steels. As shown in Figure 14c, SA508 exhibits homogeneous bainitic phase distribution. The contrast variations in the micrograph correspond to localized carbon concentration gradients, indicating carbon heterogeneity in SA508. Figure 14d shows 42CrMo has a dual-phase microstructure consisting of martensite and bainite, with phase proportions influenced by heat treatment conditions. Q235, shown in Figure 14e, exhibits a typical microstructure of banded pearlite, with a small fraction of ferrite. The microstructure of X80, illustrated in (f), exhibits a complex phase assemblage of granular bainite and polygonal/acicular ferrite phases arranged. These distinct microstructures, particularly the larger austenitic grain in size relative to other phases, highlight the correlation between microstructural diversity and variations in mechanical properties.

**Figure 15:**
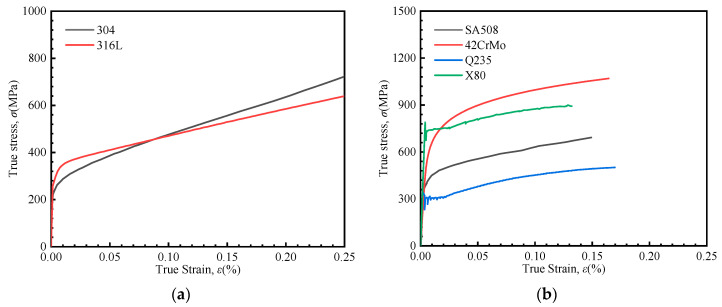
Uniaxial stress–strain curves. (**a**) SS316L and SS304, (**b**) SA508, 42CrMo, Q235 and X80.

The stress–strain curves obtained from uniaxial tensile tests are presented in Figure 15. It can be observed that SS304 and SS316 are accurately described by the linear hardening model. While SA508, 42CrMo, Q235, and X80 are more appropriately fitted using the Hollomon model.

Microstructural analysis indicates that stainless steels contain a substantial amount of austenite. This austenitic composition is likely the key factor responsible for the nearly linear hardening behavior. Conversely, microstructures comprising martensite, bainite, or pearlite typically result in power-law hardening during plastic deformation. Therefore, the Instrumented Indentation Technique (IIT) should incorporate criteria linking microstructures to appropriate constitutive models. For engineering structures, it is recommended to select a constitutive model based on the local microstructural features. The following microstructure-constitutive model selection criteria are proposed:

(1) Materials predominantly composed of austenite should be predicted using the linear hardening method.

(2) Fine-grained (martensite/bainite/pearlite, etc.) materials should be predicted using Hollomon power-law method.

Building upon these two criteria, a further extension is proposed:

(3) For materials with other microstructures, including heterogeneous ones, those with grain sizes comparable to that of austenite are more suitably predicted using the linear hardening method, while those with significantly smaller grains tend to use Hollomon power-law method.

Once the appropriate model is selected, the corresponding predictive method can be applied to derive the plastic parameter.

Although this study incorporates microstructures, it does not explicitly consider factors such as grain size. This is primarily because the focus of our work lies in understanding the macroscopic mechanical behavior of materials. Our objective is to establish a quantitative correlation between IIT and uniaxial tensile testing, rather than to investigate the influence of grain size features. Moreover, the indenter used in our tests has a diameter of 1 mm, and the maximum indentation depth is 0.1 mm, both of which are significantly larger than typical grain sizes. This indicates that our indentation experiments are conducted at the micron scale, where the response reflects bulk material behavior. Our emphasis is on capturing local mechanical property variations within engineering components, such as differences between the base metal, weld metal, and heat-affected zone (HAZ) in welded joints. The proposed IIT is designed to evaluate the local mechanical properties of these regions, rather than to resolve the influence of grain size. Therefore, within the scope of this study, it is reasonable to neglect the effects of grain size.

The observed phase evolution results from processing techniques and partly due to variations in elemental composition. Differences in elemental composition can lead to the formation of austenite or other phases, which in turn result in either linear or power-law mechanical behavior during plastic deformation. Microstructures are governed by thermo-mechanical processing parameters (e.g., the cooling rate) and variations in elemental composition (e.g., Mn, Cr, and Ni, as shown in Table 2). Alloy design principles suggest that elemental additions (e.g., Cr and Ni in SS316L/304) enhance mechanical performance through solid solutions and stacking fault energy modification. This microstructure-property relationship has been extensively validated in previous studies [5,40]. The IIT method essentially combines with microstructure effects and significantly enhances the accuracy of mechanical performance prediction, reducing errors in using a single constitutive model.

### 5.5. Experimental Verification

**Figure 16:**
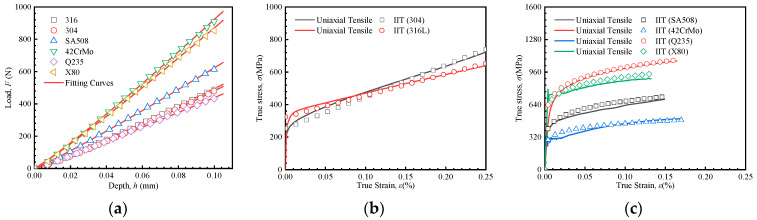
Load–depth curves and prediction results of true stress–strain curves. (**a**) Load–depth curves and fitting curves for all six materials, (**b**) prediction results of SS316L and SS304 by the IIT approach, (**c**) prediction results of SA508, 42CrMo, Q235 and X80 by the IIT approach.

Figure 16a shows the load–depth curves of indentation tests for the six metallic materials, along with the corresponding fitted curves for the loading segments. The corresponding values of *C* and *m* (for Equations (9) and (13)) are listed in Table 5. According to the IIT method, the mechanical properties of SS316L and SS304 were predicted using the linear hardening model, while those of SA508, 42CrMo, Q235, and X80 were predicted based on the Hollomon model. The predicted results are illustrated in Figure 16b,c. The stress–strain curves predicted using the proposed dual-constitutive IIT method closely match the uniaxial tensile test results for all six materials. In particular, it is noted that neither the Hollomon equation nor the linear hardening constitutive model can adequately fit the stress–strain curve of Q235. This error arises from the pronounced yield platform in Q235, a characteristic behavior not well represented by conventional constitutive models. 

**Figure 17:**
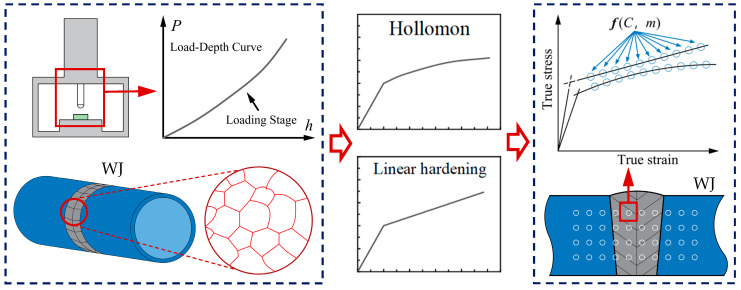
Illustration of the developed IIT.

In this study, an IIT incorporating microstructural characterization and constitutive model selection was developed. For engineering structural components, such as welded joints (WJ), the local microstructure can first be identified, as the base metal, weld metal, and heat-affected zone (HAZ) typically exhibit different microstructural features. Based on these differences, appropriate constitutive models and corresponding IIT are selected for each region. This approach enables a more accurate assessment of the distribution of mechanical properties, thereby laying the groundwork for subsequent structural integrity evaluations. As illustrated in Figure 17, the proposed method enables the non-destructive evaluation of local mechanical property distributions and potential degradation in complex structures.

Although our study focuses on macroscopic mechanical properties and assumes the materials to be isotropic, we acknowledge the potential influence of residual stresses. Several existing studies have proposed methods to derive load–depth curves of stress-free condition [41,42]. These advancements indicate that our IIT, when integrated with such techniques, could be effectively applied to welded joints. Moreover, numerous researchers have demonstrated, through uniaxial tensile tests, microstructural characterization, and indentation experiments, that surface-localized mechanical responses can reliably represent the bulk mechanical properties across different regions of welded joints [3,43,44,45].

## 6. Conclusions

In this study, the quantitative relationship between indentation responses and plastic mechanical properties was investigated using a finite element based on dual constitutive material models. A new Instrumented Indentation Technique (IIT) method was developed by integrating microstructure with constitutive models. The proposed IIT protocol was verified through experimental tests. The main conclusions are summarized as follows:

1. Different prediction methods for the mechanical properties of power-law and linear hardening materials were developed using the finite element inversion method. The predicted errors are broadly within 10%, with the maximum error at 12%.

2. Through material microstructural characterization, the relationship between the microstructure and hardening behavior was established. Austenite-dominated materials exhibit quasi-linear hardening behavior during plastic deformation, while materials with fine-grained, such as martensite, bainite, and pearlite, exhibit more power-law deformation patterns.

3. An integrated IIT method incorporating both microstructures and corresponding constitutive models was developed and applied within an engineering testing framework. The method involves selecting the appropriate constitutive model (hardening behavior) based on the dominant microstructural features, followed by the prediction of mechanical properties using corresponding formulations.

4. The reliability of the proposed IIT method was validated on six commonly used engineering steels. The results highlight the significance of selecting an appropriate constitutive model to improve the accuracy of mechanical property prediction.

This study reduces the limitations of relying on a single constitutive model and provides a fundamental reference for advancing IIT and structural integrity assessment. However, current research offers only a limited understanding of the intrinsic relationship between microstructural features and constitutive behavior. In addition, the range of material properties employed in inverse analysis remains constrained. Future studies should therefore focus on expanding the applicability of microstructure-constitutive model selection criteria and broadening the predictive range of the proposed methods. More importantly, efforts should be directed toward uncovering the fundamental correlations between microstructures and constitutive models.

## Figures and Tables

**Table 1:** Plastic parameters of materials in finite element model under two constitutive conditions.

	Yield Stress *σ*_0_ (MPa)	Strain Hardening Exponent *n* or Reduction Coefficient *δ*				
Hollomon	200	0.1	0.2	0.3	0.4	0.5
	300					
	400					
	500					
	600					
Linear hardening	200	0.004	0.006	0.008	0.010	0.012
	300					
	400					
	500					
	600					

**Table 2:** Chemical composition list (%).

	C	Si	Mn	P	S	Cr	Ni	Mo	Cu	Al	Nb + V + Ti
304	0.03	0.75	2.00	0.03	0.02	16.7	12.01	2.12	—	—	—
316L	0.08	0.75	2.00	0.045	0.02	≈19	≈10	—	—	—	—
SA508	0.226	0.194	1.375	0.007	0.001	—	—	—	0.02	0.008	—
42CrMo	0.41	0.24	0.66	0.09	0.05	1.01	0.01	0.18	—	—	—
Q235	0.14	0.19	0.34	0.03	0.034	—	—	—	—	—	—
X80	0.047	0.26	1.70	0.01	0.001	0.24	0.12	0.10	0.03	0.03	0.062

**Table 3:** Fitting coefficient values corresponding to different yield stresses in Hollomon model.

Yield Stress *σ*_0_ (MPa)	*a^H^*	*b^H^*	*d^H^*	*e^H^*	*f^H^*
200	112,878.245	−16,291.832	4258.866	0.585	0.976
300	117,946.322	−10,037.172	5162.260	0.579	0.988
400	117,779.420	−2563.466	5871.541	0.578	0.997
500	123,780.779	−119.329	7162.056	0.563	1.009
600	122,757.020	5428.674	8150.501	0.552	1.021

**Table 4:** Fitting coefficient values corresponding to different yield stresses in Linear Hardening Model.

Yield Stress *σ*_0_ (MPa)	*a* *^L^*	*b* *^L^*	*d* *^L^*	*e* *^L^*	*f* *^L^*
200	346,679.047	2554.473	−653.571	21.817	1.018
300	366,803.929	3680.230	−751.964	22.365	1.003
400	363,242.973	4905.715	−476.250	16.234	1.022
500	367,683.995	6057.947	−305.179	12.586	1.034
600	402,339.115	6943.546	−323.036	13.457	1.026

**Table 5:** Indentation response parameters of six kinds of materials.

Response Parameters	*C*	*m*
SS316L	6417.500	1.114
SS304	6742.122	1.148
SA508	7574.917	1.085
42CrMo	11,609.890	1.100
Q235	5040.309	1.061

## Data Availability

The original contributions presented in this study are included in the article. Further inquiries can be directed to the corresponding authors.

## References

[1] Singh M.P., Shukla D.K., Kumar R., Arora K.S. (2021). The structural integrity of high-strength welded pipeline steels: A review. Int. J. Struct. Integr..

[2] Turcot G., Paquet D., Lévesque M., Turenne S. (2022). A novel inverse methodology for the extraction of bulk elasto-plastic tensile properties of metals using spherical instrumented indentation. Int. J. Solids Struct..

[3] Kim J.J., Pham T.H., Kim S.E. (2015). Instrumented indentation testing and FE analysis for investigation of mechanical properties in structural steel weld zone. Int. J. Mech. Sci..

[4] Liu H., Song J., Wang H., Du Y., Yang K., Liu Y., Wang Q., Chen Q. (2021). Heterogeneous microstructure and associated mechanical properties of thick electron beam welded Ti-5Al-2Sn-2Zr-4Mo-4Cr alloy joint. Mater. Sci. Eng. A.

[5] Das G., Ghosh S., Ghosh S. (2006). Structure–property correlation of EN steel and evaluation of mechanical properties through BIT. NDT E Int..

[6] Wang K., Liao T., Xu J., Yang J., Wang P., Chen R., Qian Y., Kan Q., Li L. (2023). Identification of elastoplastic properties of materials with gradient residual stresses using a co-simulation method and nanoindentation experiments. NDT E Int..

[7] Peng W., Jiang W., Gu W., Li J., Xu T., Guo G., Zhao X., Hu X. (2025). Indentation energy method for evaluating the effect of local PWHT on pressure vessels. Int. J. Mech. Sci..

[8] Oliver W.C., Pharr G.M. (1992). An improved technique for determining hardness and elastic modulus using load and displacement sensing indentation experiments. J. Mater. Res..

[9] Tabor D. (1948). A simple theory of static and dynamic hardness. Proc. R. Soc. Lond. A.

[10] Beghini M., Bertini L., Fontanari V. (2006). Evaluation of the stress-strain curve of metallic materials by spherical indentation. Int. J. Solids Struct..

[11] Hill R., Lee E.H., Tupper S.J. (1947). The theory of wedge indentation of ductile materials. Proc. R. Soc. Lond. A.

[12] Johnson K.L. (1970). The correlation of indentation experiments. J. Mech. Phys. Solids.

[13] Park Y.J., Pharr G.M. (2004). Nanoindentation with spherical indenters: Finite element studies of deformation in the elastic-plastic transition regime. Thin Solid Film..

[14] Chen H., Cai L.X. (2016). Theoretical model for predicting uniaxial stress-strain relation by dual conical indentation based on equivalent energy principle. Acta Mater..

[15] Xue H., He J., Zhang J., Xue Y. (2021). Approach for obtaining material mechanical properties in local region of structure based on accurate analysis of micro-indentation test. China Mech. Eng..

[16] Peng J. (2012). Experimental verification of five spherical indentation-based methods in determining plastic properties of metals. Rare Metal. Mat. Eng..

[17] Haggag F.M., Byun T.S., Hong J.H., Miraglia P.Q., Murty K.L. (1998). Indentation-energy-to-fracture (IEF) parameter for characterization of DBTT in carbon steels using nondestructive automated ball indentation (ABI) technique. Scr. Mater..

[18] Hu Z., Lynne K., Delfanian F. (2015). Characterization of materials’ elasticity and yield strength through micro-/nano-indentation testing with a cylindrical flat-tip indenter. J. Mater. Res..

[19] Hu Z., Lynne K.J., Markondapatnaikuni S.P., Delfanian F. (2013). Material elastic-plastic property characterization by nanoindentation testing coupled with computer modeling. Mater. Sci. Eng. A.

[20] Zhang Y., Xue H., Wang S., Yang C., Wang Z. (2024). Study on correlation and scaling protocol between indentation stress-strain and uniaxial stress-strain. Structures.

[21] Subroto T., Miroux A., Eskin D.G., Katgerman L. (2017). Tensile mechanical properties, constitutive parameters and fracture characteristics of an as-cast AA7050 alloy in the near-solidus temperature regime. Mater. Sci. Eng. A.

[22] Samuel K.G. (2005). Limitations of Hollomon and Ludwigson stress–strain relations in assessing the strain hardening parameters. J. Phys. D Appl. Phys..

[23] Zhang Y., Xue H., Zhang S., Wang S., Sun Y., Zhang Y., Yang Y. (2022). Interaction of mechanical heterogeneity and residual stress on mechanical field at crack tips in DMWJs. Sci. Technol. Nucl. Ins..

[24] Pharr G.M., Oliver W.C., Brotzen F.R. (1992). On the generality of the relationship among contact stiffness, contact area, and elastic modulus during indentation. J. Mater. Res..

[25] Sneddon I.N. (1965). The relation between load and penetration in the axisymmetric Boussinesq problem for a punch of arbitrary profile. Int. J. Eng. Sci..

[26] Chen G.Y., Zhang X.C., Zhong J.R., Shi J., Wang Q.Q., Guan K.S. (2021). New inverse method for determining uniaxial flow properties by spherical indentation test. China Mech. Eng..

[27] Huang L.Y., Guan K.S., Xu T., Zhang J.M., Wang Q.Q. (2019). Investigation of the mechanical properties of steel using instrumented indentation test with simulated annealing particle swarm optimization. Theor. Appl. Fract. Mech..

[28] Peng W., Jiang W., Sun G., Yang B.X., Shao S., Tu T. (2022). Biaxial residual stress measurement by indentation energy difference method: Theoretical and experimental study. Int. J. Press. Vessels Pip..

[29] Hertelé S., Waele W.D., Denys R. (2011). A generic stress–strain model for metallic materials with two-stage strain hardening behaviour. Int. J. Non Linear Mech..

[30] Hradil P., Talja A., Real E., Mirambell E., Rossi B. (2013). Generalized multi-stage mechanical model for non-linear metallic materials. Thin-Walled Struct..

[31] Rasmussen K.J.R. (2003). Full-range stress–strain curves for stainless steel alloys. J. Constr. Steel Res..

[32] Chen J., Young B. (2006). Stress-strain curves for stainless steel at elevated temperatures. Eng. Struct..

[33] Chen G.Y., Zhong J.R., Zhang X.C., Guan K.S., Wang Q.Q., Shi J. (2021). Estimation of tensile strengths of metals using spherical indentation test and database. Int. J. Press. Vessels Pip..

[34] (2019). Metallic Materials—Indentation Test—Determination of Strength, Hardness and Stress-Strain Curve.

[35] Mohan S., Millan-Espitia N., Yao M., Steenberge N.V., Kalidindi S.R. (2021). Critical evaluation of spherical indentation stress-strain protocols for the estimation of the yield strengths of steels. Exp. Mech..

[36] Zhao M., Ogasawara N., Chiba N., Chen X. (2006). A new approach to measure the elastic–plastic properties of bulk materials using spherical indentation. Acta Mater..

[37] (2014). Stainless Steels—Part 2: Technical Delivery Conditions for Sheet/Plate and Strip of Corrosion Resisting Steels for General Purposes.

[38] (2009). Stainless Steel Plate, Sheet and Strip for Pressure Equipments.

[39] (2019). Metallic Materials—Tensile Testing—Part 1: Method of Test at Room Temperature.

[40] Sreepriya T., Mythili R., Reddy G.V.P. (2022). Correlation between microstructure and nanomechanical properties of 9Cr–1Mo ferritic martensitic steel through instrumented indentation technique. Mater. Sci. Eng. A.

[41] Kim J., Choi S., Lee J., Ahn H., Kim Y., Choi M., Kwon D. (2019). An indentation method for evaluation of residual stress: Estimation of stress-free indentation curve using stress-independent indentation parameter. J. Mater. Res..

[42] Peng W., Jiang W., Yang B., Sun G., Shao X. (2023). An indentation method for measuring welding residual stress: Estimation of stress-free indentation curve using BP neural network prediction model. Int. J. Press. Vessels Pip..

[43] Wu S., Xu T., Song M., Guan K. (2016). Mechanical properties characterisation of welded joint of austenitic stainless steel using instrumented indentation technique. Mater. High. Temp..

[44] Sun G., Xu F., Li G., Huang X., Li Q. (2014). Determination of mechanical properties of the weld line by combining micro-indentation with inverse modeling. Comput. Mater. Sci..

[45] Pamnani R., Karthik V., Jayakumar T., Vasudevan M., Sakthivel T. (2016). Evaluation of mechanical properties across micro alloyed HSLA steel weld joints using Automated Ball Indentation. Mater. Sci. Eng. A.

